# An Individualized Intra-Articular Stabilization Device Designed Based on 3D Printing Technology for Traumatic Instability of the Ulnohumeral Joint

**DOI:** 10.1155/2020/3056395

**Published:** 2020-11-25

**Authors:** Rong-Feng She, Yi Zhang, Bin Zhang, Yuan-Zheng Wang, Qi-Xiang Huang

**Affiliations:** Guizhou Provincial People's Hospital, Guiyang, 550002 Guizhou Province, China

## Abstract

We aimed to design an individualized intra-articular stabilization device based on 3D printing technology and investigate the clinical effects of this device for treating traumatic instability of the ulnohumeral joint. This study enrolled nine patients with traumatic instability of the ulnohumeral joint (age: 47.2 ± 1.80 years) who received treatment between March 2018 and March 2019 in our hospital. All patients underwent a thin-layer computed tomography (CT) scan of the elbow before surgery. The original injury and repair models of the elbow were printed using 3D printing technology based on CT data. An individualized intra-articular stabilization device was designed with a 2.0 mm Kirschner wire based on the repair model. Nine patients agreed to receive surgical treatment for elbow disease and placement of the intra-articular stabilization device. The nine patients underwent open reduction through a posterior median approach, and the intra-articular stabilization device was placed in the elbow. Operation time, intraoperative blood loss, and postoperative complications were recorded and followed up. The device was removed at two postoperative months, and the Mayo score was used to evaluate elbow function. Four months after removing the intra-articular stabilization device, elbow joint function was evaluated again using the Mayo score. The mean operation time was 100.1 ± 8.2 min, and the mean intraoperative blood loss was 35.5 ± 7.1 ml. No complications occurred after operation. Two months after surgery, eight patients received an excellent Mayo score, and one patient received a good Mayo score. Four months after removal of the intra-articular stabilization device, eight patients received an excellent Mayo score, and one patient received a good Mayo score. The individualized intra-articular stabilization device can increase ulnohumeral stability and achieve rapid functional recovery of the elbow.

## 1. Introduction

The elbow is composed of bones and ligaments, and the ulnohumeral joint primarily participates in the flexion and extension of the elbow. Thus, ulnohumeral instability causes elbow instability and dysfunction [1]. Clinically, ulnohumeral instability is commonly caused by trauma, such as posterior dislocation of the elbow joint and terrible triad of the elbow. In addition to reconstruction of the structural integrity of the bones and ligaments, the main methods to improve elbow instability are generally the combination of a hinged external fixator or external fixation with a plaster cast and elbow brace [2, 3]. A hinged external fixator with an elbow brace has achieved good clinical results for treating elbow instability. However, the radial nerve may be damaged during implantation of a hinged external fixator, which may cause inconveniences in the patients' life and needle tract infection [4]. Moreover, external fixation with a plaster cast or elbow brace does not allow early functional exercise and may result in elbow stiffness [5]. To solve the aforementioned problems, Orbay et al. [6, 7] used a 2.5 mm Kirschner wire to design an internal joint stabilizer to treat elbow instability during the operation. A multicenter randomized controlled study concluded that this method not only allows elbow movement, but it also increases elbow stability and has a good clinical effect. Pasternack et al. [8] used an internal joint stabilizer to treat traumatic elbow instability and achieved good clinical effects. Thus, an internal joint stabilizer is a good choice for treating traumatic elbow instability. Therefore, the authors used 3D printing technology to print a repair model of the elbow injury. Based on the 3D repair model, an individualized intra-articular stabilization device was designed *in vitro* to reduce the time spent shaping the intra-articular stabilization device during the operation, reduce surgical trauma, improve the degree to which the intra-articular stabilization device is individualized to the patient, and increase the consistency between the intra-articular stabilization device and the elbow rotation center. Clinical studies have indicated that 3D printing technology is used to assist in designing new internal stabilization devices, which can increase ulnohumeral stability, allow the elbow to move along the elbow rotation center, enable the elbow to perform exercises earlier, and achieve rapid rehabilitation after elbow joint injury.

## 2. Subjects and Methods

### 2.1. Subjects

This study enrolled nine patients with traumatic instability of the ulnohumeral joint who received treatment between March 2018 and March 2019 in our hospital. These patients included three cases of three- to nine-week-old dislocations and six cases of terrible triad of the elbow (fresh fracture, posterior dislocation). The nine patients included six males and three females, aged 28–60 years (mean age: 47.2 ± 1.80 years). Of the cases of terrible triad of the elbow, three were type II and three were type III according to the Mason classification of radial head fracture, whereas four were type I and two were type II according to the O'Driscoll classification of ulnar coronoid process fracture. The operation was performed after the relevant preoperative examinations were conducted and absolute contraindications were eliminated. The implantation of the intra-articular stabilization device was approved by the Ethics Committee of Guizhou Provincial People's Hospital, China. All patients provided written informed consent.

### 2.2. Inclusion Criteria

The inclusion criteria were as follows: (1) history of trauma, (2) imaging evidence of ulnohumeral dislocation, (3) closed injury, (4) no vascular or nerve rupture in the elbow, and (5) no history of psychosis and good compliance.

### 2.3. Exclusion Criteria

The exclusion criteria were as follows: (1) obvious congenital malformation in the elbow; (2) pathological fracture; (3) imaging showing humeral condyle, olecranon fracture of the ulna, and dislocation of the proximal radioulnar joint; and (4) the elbow injury was combined with vascular and nerve rupture.

### 2.4. Design of the Intra-Articular Stabilization Device

#### 2.4.1. Data Acquisition and Modeling

The injured elbow underwent a thin-layer CT scan. The 3D printing of the original damage model was performed with the CT scan data at a 1 : 1 ratio. Simultaneously, the computer was used to simulate the repair of the injured elbow, and 3D printing technology was used to print out the repair model. The software for processing CT scan DICOM data used was Mimics 19, and the 3D printing software used was Simplify 3D 3.0.

#### 2.4.2. Design of the Intra-Articular Stabilization Device and the Individualized Preshaping before Operation

The repair model was used as the template for the intra-articular stabilization device. A 2.0 mm Kirschner wire was used to shape the intra-articular stabilization device. The rotation center of the medial and lateral condyles of the distal humerus was the center of the rotation axis of the intra-articular stabilization device. The device was inserted into the medial condyle via the lateral condyle of the humerus and was shaped along the normal anatomical structure of the posterior elbow. Finally, the intra-articular stabilization device was shaped into an “8” shape that could pass through 3.5 mm screws and was fixed to the olecranon with 3.5 mm screws. Before the operation, the individualized intra-articular stabilization device was preshaped, and the repair model was disinfected for standby.

### 2.5. Surgical Methods

#### 2.5.1. Surgical Methods for Chronic Dislocation of the Elbow

Using a posterior median approach, the elbow was still dislocated but the proximal radioulnar joint was not dislocated during the operation. The elbow joint cavity was exposed through both sides of the triceps tendon to clean up the soft tissue of ectopic ossification and hyperplasia. After reduction, the elbow's range of motion was satisfactory, but the stability was poor. A 1.5 mm Kirschner wire was inserted into the medial condyle through the rotation center of the humeral condyle. The Kirschner wire was located in the rotation center of the humeral condyle under C-arm fluoroscopy. Then, 2.0 and 2.5 mm Kirschner wires were gradually used to drill and form the rotation center. The individualized intra-articular stabilization device that was designed and shaped before the operation was inserted and fixed on the olecranon with two 3.5 mm screws. The movement track of the elbow was normal during passive movement. The range of motion of the elbow was between 0 and 140°. The stability of the elbow was good. There was no obvious compression between the intra-articular stabilization device and the ligament and joint capsule around the elbow. The injured lateral collateral ligament was reconstructed with a suture anchor. The incision was washed and sutured after the drainage tube was placed in the operation area. The three patients with an old dislocation only underwent repair of the lateral collateral ligament; they did not receive treatment for the medial collateral ligament.

#### 2.5.2. Surgical Methods of Terrible Triad of the Elbow

The anesthesia, body position, tourniquet, sterilizing, and draping methods were the same as those used for the patients with old dislocations. Using the posterior median approach, the ulnar nerve was separated and protected. The radial head was exposed through the space between the anconeus muscle and the extensor carpi ulnaris. Reduction and internal fixation or radial head replacement were performed according to the radial head fracture. Of the six cases, one was fixed using a countersunk screw, three using a plate screw, and two by radial head replacement. After reduction, the range of motion of the elbow was satisfactory but its stability was poor. The intra-articular stabilization device was inserted in the same method as that used for an old dislocation. The injured lateral collateral ligament was repaired with a suture anchor. The coronoid process fracture and medial collateral ligament were not treated specially. The coronoid process fractures of the ulna in this group were classified as the O'Driscoll Type I or II. The placement of the intra-articular stabilization device in the elbow significantly increased elbow stability; thus, the coronoid process fracture fragments were not treated. The incision was washed and sutured after the drainage tube was placed in the operation area.

The nine patients were treated and followed up by the same group of surgeons.

### 2.6. Postoperative Treatment

After the operation, analgesia was administered, drainage tubes with a drainage volume of <10 ml in 24 h were removed, and treatments to prevent infection and heterotopic ossification were performed. Twenty-four hours after the operation, the elbow was first exercised passively. Active and passive functional exercises were performed after removal of the drainage tube. The patients returned to the hospital for reexamination at two weeks, four weeks, and two months postoperatively. The intra-articular stabilization device was removed two months after the operation based on elbow recovery status.

### 2.7. Postoperative Observation Indicators

Operation time, intraoperative blood loss, and postoperative complications were recorded and followed up. Complications included foreign body sensation, infection, heterotopic ossification, injuries to the radial and ulnar nerves, and loosening, falling off, and rupture of the intra-articular stabilization device. Two months after placement of the device, the Mayo score was used to evaluate elbow function [6]. Elbow function was reevaluated using the Mayo score four months after device removal.

### 2.8. Statistical Analysis

All data were analyzed using SPSS 19.0 software. The data displayed a normal distribution. Measurement data are expressed as the mean ± standard deviation. Differences between groups were compared with a one-way analysis of variance and the least significant differences test. A value of *P* < 0.05 was considered statistically significant.

## 3. Results

Nine patients were followed up for 13.2 ± 1.0 months. The operation time ranged from 60 to 130 min (mean 100.1 ± 8.2 min). The intraoperative blood loss ranged from 20 to 60 ml (mean 35.5 ± 7.1 ml). No foreign body sensation, infection, heterotopic ossification, or ulnar nerve injury were reported in the nine patients. There was no loosening, falling off, or rupture of the intra-articular stabilization device in the follow-up period at two months after placement.

The elbow joint underwent a functional evaluation after the operation. Two months after the intra-articular stabilization device was placed, the mean elbow flexion and extension motion range was 123.7 ± 5.4°, and the mean forearm rotation motion range was 145.9 ± 7.5°. Eight patients reported an excellent Mayo score, and one patient had a good Mayo score. Four months after the intra-articular stabilization device was removed, the mean elbow flexion and extension motion range was 124.2 ± 5.8°, and the mean forearm rotation motion range was 147.1 ± 7.1°. Eight patients reported an excellent Mayo score, and one patient had a good Mayo score. There were no significant differences in elbow motion range or Mayo score after the two operations (*P* > 0.05) (Table 1).

There were no significant differences in the range of flexion and extension, forearm rotation range, and Mayo score two months after inserting the device and four months after removing the device (*P* > 0.05).

### 3.1. Typical Cases

#### 3.1.1. Case 1

**Figure 1:**
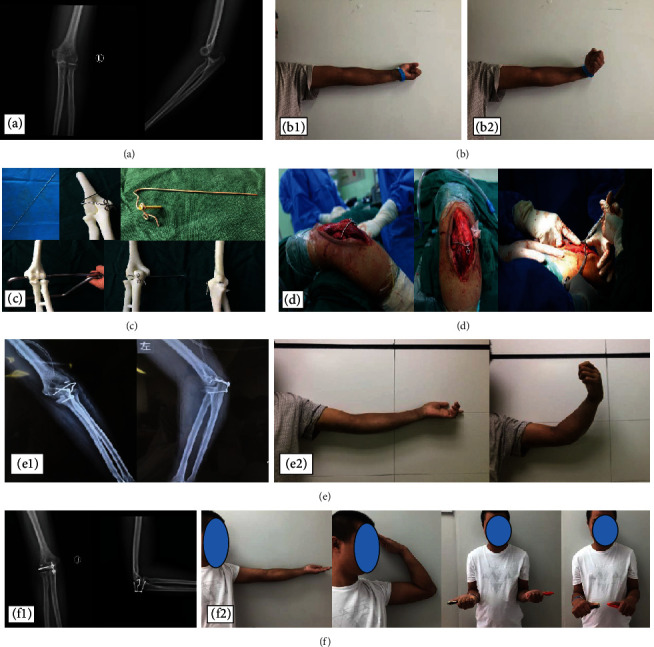
(a) X-ray on admission of a 28-year-old man showing a dislocated left elbow joint. (b-1) Extension function of the elbow. (b-2) Flexion function of the elbow. (c) Individualized design of the intra-articular stabilization device based on the 3D model of the elbow joint injury and repair. (d) Placement of the intra-articular stabilization device. (e-1) X-ray films immediately after placement. (e-2) Active flexion and extension five days after surgery. (f-1) X-ray films two months after placement show that the elbow joint alignment is normal, and the device has not become loose, fallen off, or broken. (f-2) Elbow extension, elbow flexion, forearm supination, and forearm supination functions two months after placement.

**Figure 2:**
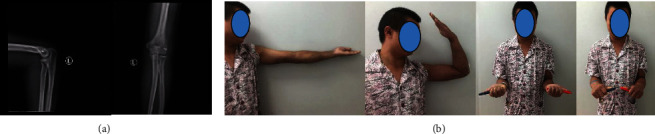
(a-1) X-ray films immediately after removal show that the elbow joint alignment is normal without dislocation. (a-2) Elbow extension function, flexion function, forearm supination function, forearm pronation function, and good stability of the elbow joint five days after removal.

A 28-year-old male patient was admitted to the hospital because of pain in the left elbow caused by falling and limited movement for more than two months. On admission, the patient was diagnosed with an old dislocation of the left elbow and stiff elbow. The following surgical method was followed: the left dislocated elbow underwent open reduction. After cleaning the joint, the intra-articular stabilization device was placed, and the lateral collateral ligament was repaired with a suture anchor (Figures 1 and 2).

#### 3.1.2. Case 2

**Figure 3:**
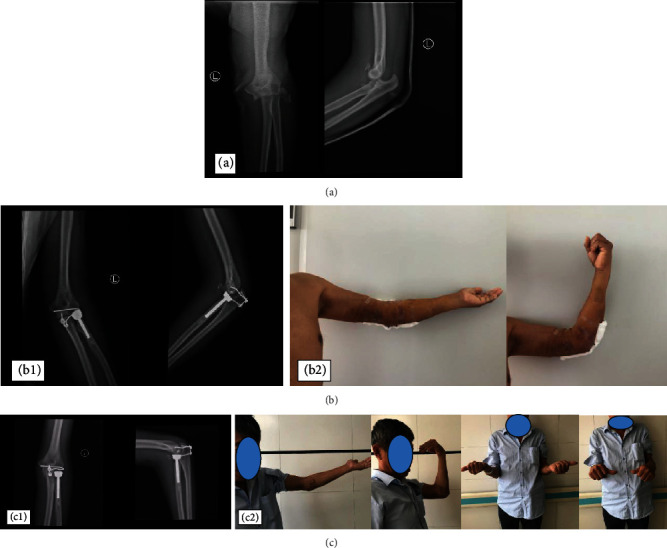
(a) X-ray films of the left elbow show dislocation of the left elbow, comminuted fracture of the radial head, and fracture of the coronoid process. (b-1) X-ray films five days after placement of the device show that the left dislocated elbow has been reduced, the radial head has been replaced, and the device has been placed. (b-2) Active flexion and extension five days after placement. (c-1) X-ray films two months after placement show that the elbow joint alignment is normal, and the device has not become loose, fallen off, or broken. (c-2) Elbow extension function, flexion function, forearm supination function, and forearm pronation function two months after placement.

**Figure 4:**
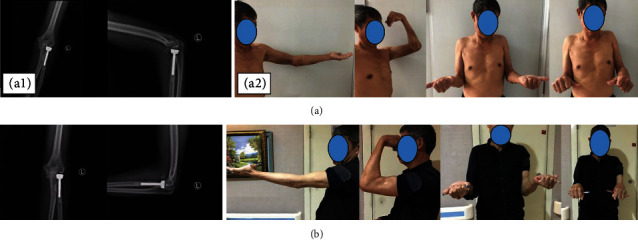
(a-1) X-ray films one month after removing the device show that the elbow joint alignment is normal, and there are no abnormalities such as dislocation. (a-2) Elbow extension function, elbow flexion function, forearm supination function, forearm pronation function, and good elbow stability one month after removal. (b-1) X-ray films four months after removal show that the elbow joint alignment is normal, and the radial head prosthesis is not loose or falling off. (b-2) Elbow extension function, elbow flexion function, forearm supination function, forearm pronation function, and good elbow stability four months after removal.

A 60-year-old male patient was admitted to the hospital because of “pain and distention of the left elbow caused by falling and limited activity for seven days.” The patient was diagnosed with terrible triad of the left elbow. The surgical method was conducted as follows: the left dislocated elbow underwent open reduction and radial head replacement. The intra-articular stabilization device was inserted, and the lateral collateral ligament was repaired with a suture anchor. The fracture of the coronoid process of the ulna was classified as the Regan-Morrey type II, which was not treated (Figures 3 and 4).

## 4. Discussion

Elbow instability leads to elbow dysfunction, which causes inconveniences in both the work and life of patients. Different clinical methods are used to treat elbow instability, including plaster support, external brace fixation, and external bracket fixation. Although these methods have achieved good clinical effects, they have some shortcomings. To avoid the shortcomings of the above methods, the authors used 3D printing technology to design an individualized intra-articular stabilization device of the elbow to treat elbow instability. Observations of nine patients who received this device demonstrated that the device has a good clinical curative effect. Traumatic instability of the ulnohumeral joint is common in patients with elbow dislocation and terrible triad of the elbow. Most patients with acute elbow dislocation can obtain a good curative effect by manual reduction and external fixation with a plaster cast in the early stages [9]. Nevertheless, unstable elbow dislocation often leads to a missed diagnosis or recurrent dislocation. A previous study reported that the rate of missed diagnosis was 15–30% [10], which may eventually lead to old dislocation of the elbow and the need for reconsultation due to elbow dysfunction. Currently, old elbow dislocations are not commonly observed in the clinic, but it is very difficult for clinicians to treat this condition, and the curative effect is uncertain. A previous study showed that elbow dislocations should be operated on within three months to obtain an acceptable curative effect [11]. For patients with obsolete elbow dislocation within the past three months, most doctors recommend surgical treatment combined with a hinged external fixator for good clinical results [12, 13]. It is difficult to treat terrible triad of the elbow. Some treatments have good therapeutic effects, and some have poor therapeutic effects; however, there is no ideal treatment. Nevertheless, most scholars still advocate for surgical treatment [14]. During the follow-up of this condition, 12% of patients experienced elbow stiffness after trauma [15]. To increase elbow stability and perform early functional exercises for patients with terrible triad of the elbow, some scholars advocate the combined use of a hinged external fixator. Motisi et al. [16] found that a hinged external fixator effectively maintained elbow stability, prevented elbow dislocation, and achieved a satisfactory clinical effect in 35 patients with terrible triad of the elbow.

Some patients with an old dislocation treated by open reduction and terrible triad of the elbow treated by surgery may require a hinged external fixator. This method has achieved a satisfactory clinical effect, but the technical requirements for the placement of the hinged external fixator in the elbow are high. Simultaneously, there is a risk of injury to the radial nerve and iatrogenic ulnar fracture during implantation. Adverse factors such as needle tract infection, external fixation needle loosening, inconvenience to patients' daily life, and nursing care (strengthen the care of the needle path of the external fixator after the operation to prevent needle path infection) may occur after surgery [17]. Moreover, a hinged external fixator is relatively expensive. Therefore, certain researchers hope to increase the stability of the elbow using an internal fixator to avoid the adverse effects of using an external fixator. Jorge et al. [6] used a 2.5 mm Kirschner wire to design an elbow stabilizer for elbow instability. The results of Jorge et al. [6] indicated that this technology restores elbow stability and mobility. For patients with severe elbow instability, this seems to be a promising treatment, but the device must be removed by a second operation. Thus, to increase elbow stability, allow the injured elbow to be exercised early, and avoid the complications caused by an external fixator, the authors designed an individualized intra-articular stabilization device. To make the device more suitable for individual differences during the operation and to utilize the advantages of 3D printing technology in the orthopedic field, the repair model was printed using 3D printing technology at a 1 : 1 ratio before the operation. The intra-articular stabilization device was designed before the operation based on the repair model. During the operation, only minor adjustment is necessary to place the device, reducing operation time and blood loss. Clinical case results showed that the operation time in nine patients ranged between 60 and 130 min. Blood loss results showed that due to the use of a tourniquet, bleeding was reduced, and the intraoperative blood loss was 20–60 ml. Elbow exercises could be gradually performed immediately after the operation. Therefore, the author's experience in using the intra-articular stabilization device demonstrated that the device can increase elbow stability, prevent recurrent elbow dislocation, and allow for early functional exercise. However, the preoperative plan should be clearly established. The individualized design of the intra-articular stabilization device should be based on the elbow repair model and created before the operation. The rotation center of the elbow can be more easily and accurately determined under direct vision during the operation. The intra-articular stabilization device can be fixed with only two screws, which can effectively reduce operation time and trauma, and the cost is relatively less. However, whether the medial collateral ligament of the elbow is repaired remains controversial. Gong et al. [18] found that only by repairing the lateral collateral ligament can one obtain a better clinical effect than by repairing the medial and lateral collateral ligaments during the surgical treatment of terrible triad of the elbow. In this study, the medial collateral ligament of the elbow was not repaired, and the elbows of the nine patients obtained good stability after surgery. It must also be determined whether a fracture of the coronoid process should be fixed in patients with terrible triad of the elbow. Papatheodorou [19] did not perform special treatments for the Regan-Morrey type I or type II coronoid process fractures after reconstruction of the radial head and LUCL when treating patients with terrible triad of the elbow. The postoperative curative effect indicated that the elbow still maintained good stability. Antoni et al. [20] found that in 30 patients with terrible triad of the elbow, the coronoid process fracture was fixed in 11 cases but not in 19. The 54-month follow-up results showed that there was no significant difference in elbow function and imaging between the two groups. Therefore, there are still different opinions on whether type I or II coronoid process fractures must be fixed. In this group of cases, the O'Driscoll Type I or II coronoid process fractures were not fixed after placing the intra-articular stabilization device, which reduced the operation time, blood loss, and surgical trauma. Furthermore, postoperative follow-up demonstrated that the elbow maintained good stability and satisfactory function after removal of the device, which can guide us to avoid fixing coronoid process fractures in terrible triad patients with type I or II coronoid process fractures after the placement of the intra-articular stabilization device. However, given the small number of cases in this group, more clinical cases and long-term follow-up are necessary to confirm this conclusion. These findings can at least provide an idea and method for clinical practice.

The postoperative follow-up of the nine patients confirmed that there was no obvious foreign body sensation after placing the intra-articular stabilization device, which may be related to the good match between the device and the anatomical structure of the elbow. This requires adequate preoperative planning. Simultaneously, no infections occurred, which indicates that the device has a good application prospect. However, the above results may also be related to the small number of cases and the short follow-up time. The nine patients did not suffer from ectopic ossification of the elbow after operation, which may be related to the oral drugs provided for ectopic ossification and adequate drainage after the operation. There was no ulnar nerve injury after the placement of the device, which may be related to the placement of the device under direct vision during the operation and the need for the device to be placed 2 mm below the bone surface at the exit of the medial condyle of the humerus. No loosening, falling off, or rupture was identified during the follow-up, which indicates that the intra-articular stabilization device had good stability in the body. This may also be related to the removal of the device two months after surgery, the short retention time in the body, and the small number of cases, in addition to the good toughness and rigidity of the Kirschner wire. There was no significant difference in elbow flexion and extension range, rotation range, and Mayo score two months after implantation and four months after removal of the device, which may be associated with the elbow functional exercises performed immediately after device implantation. This demonstrates that the intra-articular stabilization device has no obvious hindrance to elbow movement and that the elbow maintains good stability after removal of the device. Therefore, the device can provide early elbow stability, allow early elbow movement, and create a good healing environment for elbow fracture and ligament repair.

## 5. Conclusion

In summary, a 3D model of the elbow repair can be used to design an individualized intra-articular stabilization device for ulnohumeral instability caused by elbow dislocation or terrible triad of the elbow. The placement of this device in the elbow can increase ulnohumeral stability and allow early functional elbow exercises, has a good clinical effect, and results in rapid functional rehabilitation of the elbow. Compared with external fixation with plaster support, external brace fixation, and a hinged external fixator, the application of an intra-articular stabilization device can effectively reduce the incidence of elbow stiffness. It is relatively easy to place the intra-articular stabilization device during the operation, which reduces the risk of radial nerve injury and ulnar iatrogenic fracture; this also avoids the risk of infection or loosening of the needle tract after placement of the external fixation bracket and improves the patient's postoperative comfort. Compared with other scholars' intra-articular stabilization devices, the authors used 3D printing technology to design an individualized intra-articular stabilization device in vitro. This reduces the time spent shaping the intra-articular stabilization device during the operation, reduces surgical trauma, improves the extent to which the intra-articular stabilization device matches with the elbow, and increases the consistency between the intra-articular stabilization device and the elbow rotation center. However, the device is only suitable for patients with ulnohumeral instability. Other suitable treatments are required if the patient simultaneously has dislocation of the proximal radioulnar joint or a fracture of the olecranon and humeral condyle. The intra-articular stabilization device is shaped by a 2.0 mm Kirschner wire, and the device is slightly weaker than that of an internal elbow stabilizer made of internal fixation material; nevertheless, Kirschner wire is cheap. The device requires removal via a second operation. Given the small number of cases and short follow-up time in this study, clinical applications and related complications of the intra-articular stabilization device must be confirmed with more clinical cases and long-term follow-up.

## Figures and Tables

**Table 1:** Functional evaluation of the elbow joint after operation (*X* ± *S*).

Time	Range of flexion and extension	Forearm rotation range	Mayo score	
			Excellent	Good
2 months after insertion of the device	123.7 ± 5.4°	145.9 ± 7.5°	8	1
4 months after removing the device	124.2 ± 5.8°	147.1 ± 7.1°	8	1

## Data Availability

The data used to support the findings of this study are available from the corresponding author upon request.
